# Photocontrolled dissociation and toehold-mediated strand displacement-based synergistic regulation of CRISPR-Cas12a

**DOI:** 10.1093/nar/gkaf1178

**Published:** 2025-11-08

**Authors:** Jia-ni Wu, Changjiang Li, Zhujun Liu, Xiaolong Li, Jiarun Wang, Xiaoding Lou, Fan Xia, Jun Dai, Tongbo Wu

**Affiliations:** School of Pharmacy, and Department of Obstetrics and Gynecology, National Clinical Research Center for Obstetrics and Gynecology, Tongji Hospital, Tongji Medical College, Huazhong University of Science and Technology, Wuhan 430030, China; School of Pharmacy, and Department of Obstetrics and Gynecology, National Clinical Research Center for Obstetrics and Gynecology, Tongji Hospital, Tongji Medical College, Huazhong University of Science and Technology, Wuhan 430030, China; School of Pharmacy, and Department of Obstetrics and Gynecology, National Clinical Research Center for Obstetrics and Gynecology, Tongji Hospital, Tongji Medical College, Huazhong University of Science and Technology, Wuhan 430030, China; School of Pharmacy, and Department of Obstetrics and Gynecology, National Clinical Research Center for Obstetrics and Gynecology, Tongji Hospital, Tongji Medical College, Huazhong University of Science and Technology, Wuhan 430030, China; School of Pharmacy, and Department of Obstetrics and Gynecology, National Clinical Research Center for Obstetrics and Gynecology, Tongji Hospital, Tongji Medical College, Huazhong University of Science and Technology, Wuhan 430030, China; State Key Laboratory of Biogeology and Environmental Geology, Faculty of Materials Science and Chemistry, China University of Geosciences, Wuhan 430074, China; State Key Laboratory of Biogeology and Environmental Geology, Faculty of Materials Science and Chemistry, China University of Geosciences, Wuhan 430074, China; School of Pharmacy, and Department of Obstetrics and Gynecology, National Clinical Research Center for Obstetrics and Gynecology, Tongji Hospital, Tongji Medical College, Huazhong University of Science and Technology, Wuhan 430030, China; School of Pharmacy, and Department of Obstetrics and Gynecology, National Clinical Research Center for Obstetrics and Gynecology, Tongji Hospital, Tongji Medical College, Huazhong University of Science and Technology, Wuhan 430030, China

## Abstract

The *trans*-cleavage activity of the CRISPR-Cas system holds broad potential across diverse fields, yet precise spatiotemporal regulation remains challenging due to the predominantly single-direction control strategies available. Here, we present a bidirectional, multi-round modulation strategy for CRISPR-Cas12a *trans*-cleavage activity, utilizing toehold-mediated strand displacement and photocontrolled dissociation. This approach enables dynamic transitions between on and off states: Cas12a activity is activated by an activator, inhibited by a photosensitive blocker, and reactivated via UV light. We further integrated this system with DNA cryptography, establishing a hierarchical temporal authorization system that enhanced cryptographic security. The method supported multi-round modulation, achieving restoration of 95.4% activity after multiple cycles in the on state while maintaining suppression to 12.4% in the off state. This precise control strategy provides a versatile tool for spatiotemporal regulation in CRISPR-based applications, with significant implications for advanced gene editing, diagnostics, and bioengineering.

## Graphical Abstract

**Graphical Abstract:**
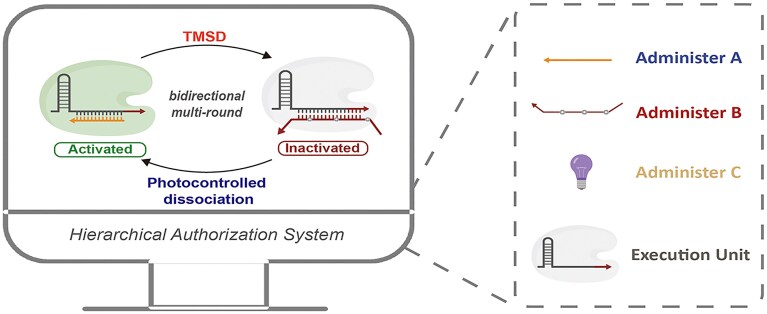

## Introduction

The clustered regularly interspaced short palindromic repeats (CRISPR) system is a crucial component of the prokaryotic adaptive immune system, defending against invasive nucleic acids through complement-dependent recognition and cleavage events mediated by guide RNAs (gRNAs) or CRISPR RNAs (crRNAs) [1]. Today, it serves as a prominent gene editing tool, enabling precise gene regulation through the specific pairing of crRNA and DNA sequences within the CRISPR-Cas system [2, 3]. The CRISPR-Cas system also exhibits *trans*-cleavage nuclease activity, and its programmability and reactivity have contributed to its widespread application in fields of molecular diagnostics, such as SHERLOCK [4], DETECTR [5], CONAN [6], and others [7–9].

Despite the significant potential of the CRISPR-Cas system in biosensing and molecular diagnostics, the non-specific nature of its *trans*-cleavage activity can lead to unintended cleavage when the CRISPR-Cas remains activated in the reaction system, causing irreversible damage to other system components [10]. Therefore, it remains crucial to precisely regulate the cleavage activity of CRISPR-Cas, construct a sensitive response system, and advance the development of more accurate and controllable CRISPR-Cas tools [11]. Such improvements will enhance the system’s utility, enabling deeper exploration and broader application of CRISPR technology.

**Figure 1.**
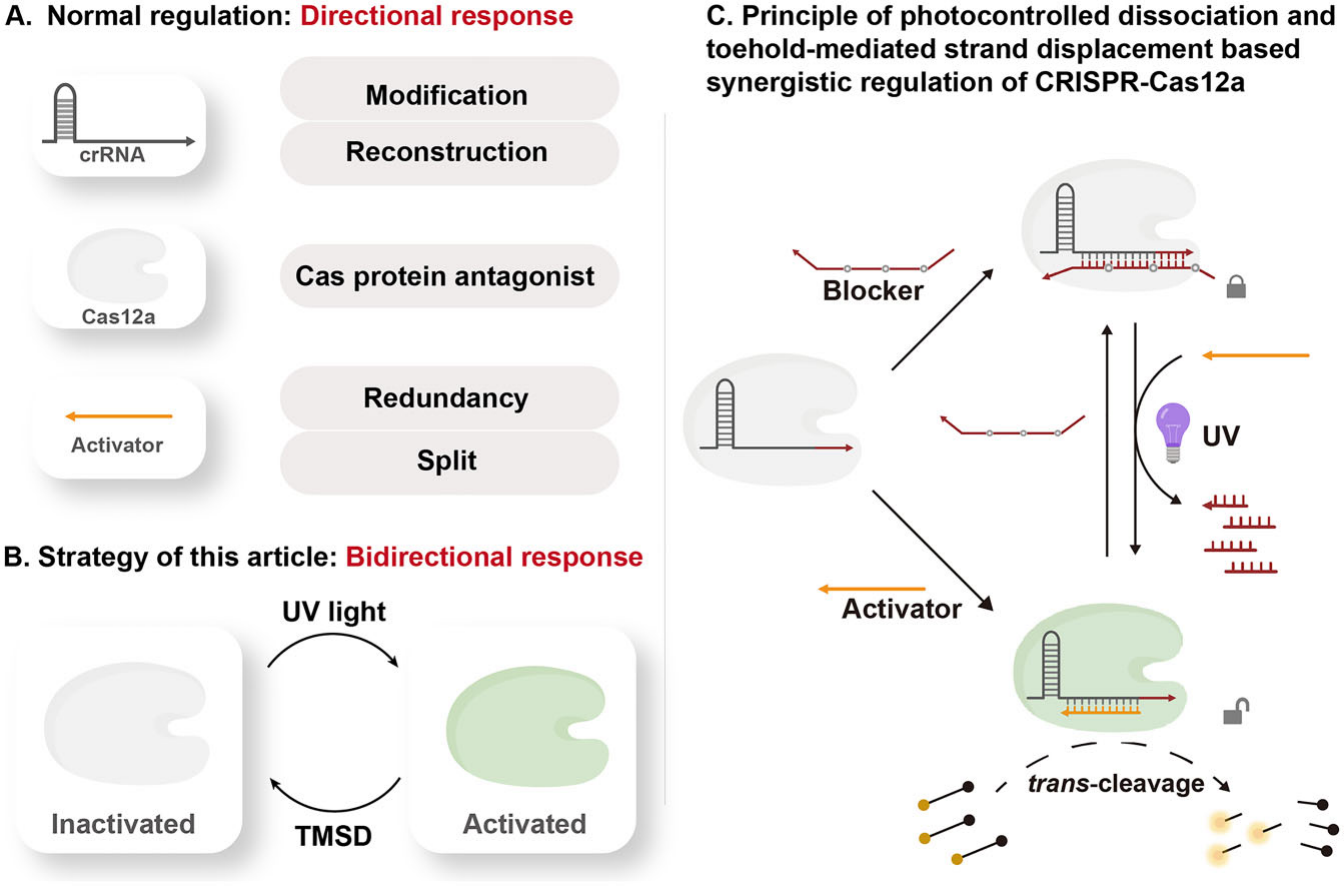
Principle of this strategy. (**A**) Modulation of Cas12a *trans*-cleavage activity by altering different aspects of crRNA, Cas12a. (**B**) Modulation of Cas12a *trans*-cleavage activity by using TMSD and photocontrolled dissociation. (**C**) Detailed principle of Cas12a activity regulation proposed by the manuscript.

Cas12a is one of the most widely used proteins in the CRISPR-Cas system due to its high *trans*-cleavage activity [12]. Current strategies to modulate Cas12a *trans*-cleavage activity include crRNA chemical modification [13, 14], crRNA structural modulation [15], the structure of DNA activators [16, 17], and the use of Cas protein antagonists [18]. Among these, crRNA-centered regulation is the most common approach. Existing literature primarily describes two strategies for crRNA-based regulation: irreversible activation via light stimulation [19] and reversible control through external ligands [20] (Fig. 1A); however, activation through light-sensitive motifs or structure-blockers in crRNA is typically unidirectional, limiting the ability to halt Cas protein cleavage at a precise time. Cas protein antagonists, which serve as activity switches, can inhibit Cas protein function by attaching a linker segment to the protein. However, this method requires substantial technical knowledge and resources, as well as the use of other enzymes to cleave the linker. Additionally, once the linker is cleaved, reconnecting the Cas protein and its antagonist is difficult, and this method typically only allows unidirectional regulation (off to on). Some studies have also explored using chemical agents to regulate crRNA by adding reducing or oxidizing agents, thereby altering the crRNA’s state [21]. Although this method enables reversible control of Cas protein activity (off to on to re-off), reactivation after the final OFF state is challenging, and the use of complex reagents limits its applicability. Regarding the regulation of DNA activators, the majority have primarily focused on property exploration, with only a limited number being applied to modulate Cas12a activity. The exploration of switch-based regulation for DNA activators remains a vast area for further investigation. Overall, there remains a need for simpler, more precise spatiotemporal control of Cas protein function to enable flexible applications across diverse scenarios.

The toehold-mediated strand displacement (TMSD) reaction is widely applied in dynamic nucleic acid nanotechnology [22, 23]. During strand displacement, the invading strand binds first to the toehold region of a double-stranded substrate, initiating an invasion that displaces one strand of the original duplex [24]. This process forms a new double-stranded structure and reaches a stable, thermodynamically equilibrated state. In recent years, TMSD has emerged as a significant methodology for regulating biologically related reactions both *in vivo* and *in vitro* [25]. Previous studies have demonstrated the feasibility of integrating strand displacement into the CRISPR-Cas system: a short RNA strand binds to the crRNA in the ribonucleoprotein (RNP) complex, allowing the activator to dynamically regulate Cas protein nuclease activity through the TMSD process [26] and a short RNA strand binds to the target strand (TS) of Cas protein in the RNP complex, allowing the crRNA displace the original short RNA *in situ* imaging [27].

In response to these considerations, we developed a strategy to achieve bidirectional, multi-round modulation of the *trans*-cleavage activity of CRISPR-Cas12a, enabling transitions from on to off and back on through a combination of TMSD and photocontrolled dissociation (Fig. 1B). We subsequently integrated this system with a DNA cryptography strategy to develop a hierarchical temporal authorization system, improving the security of DNA cryptography for potential use in complex biochemical reactions.

## Materials and methods

### Chemicals and materials

All oligonucleotides used in this study (sequences in Supplementary Table S1) were synthesized and purified by Sangon Biotech Co., Ltd (Shanghai, China). EnGen Lba Cas12a (Cpf1, LbCas12a) and 10 × NEBuffer 4 (the composition of the diluent and buffer is shown in Supplementary Table S2) were brought from New England Biolabs Inc (Beijing, China). Deionized water (DNase/RNase free) was obtained from Tiangen Biotech (Beijing, China) and used in all experiments. The concentrations of DNA/RNA oligonucleotides were measured by NanoDrop 2000 UV-vis Spectrophotometer (ThermoFisher Scientific, MA, USA). Q1600 Real-Time Fluorescence PCR Instrument (Boheng Technology Co., Hangzhou, China) was used to prepare the double strand. Fluorescence was detected using the Rotor-Gene Q real-time PCR instrument (QIAGEN, Hilden, Germany). The UV flashlight was obtained from SHENYU Lighting Technology Co (Ningbo, China).

### Preparation of dsDNA (Normal activator/ spDNA/ pcDNA)

In a 200 μL PCR tube, normal activator/spDNA/ pcDNA (1.6 μL, 25 μM), complementary chains (1.9 μL, 25 μM), and NEBuffer 4 (2 μL, 10×) were combined, and the total volume was adjusted to 20 μL with deionized water. The mixture was heated to 95°C for 1 min, then cooled to 65°C at 6°C/min for 1 min, followed by cooling to 55°C at 6°C/min for 1 min, and finally cooled to 37°C at 6°C/min to complete annealing. The double-stranded complex (non-phosphorothioate-modified and phosphorothioate-modified) preparation process used to explore the double-stranded switch is the same as the above. The double-stranded complex was then stored at 4°C. The procedure was performed on the Q1600 Real-Time Fluorescence PCR Instrument.

### Exploration of the inhibitory effects of intermediate modifications

In a 200 μL PCR tube, 0.4 μL of LbCas12a (1 μM), 2 μL of crRNA (200 nM), and 2 μL of NEBuffer 4 (10×) were combined, and the volume was adjusted to 17 μL with deionized water. The mixture was incubated at 37°C for 15 min. Then, 2 μL of HEX-ssDNA reporter (2.5 μM) and 1 μL of activator or ss-spDNA (single modified) were added to reach a final volume of 20 μL. Fluorescence intensity was immediately measured at 37°C using the Rotor-Gene Q real-time PCR instrument, with an excitation wavelength of 558 nm and an emission wavelength of 533 nm for the hexachlorofluorescein (HEX) channel (gain = 5). The Spacer C6 modifications in various spDNAs are located at positions + 1 to + 17, respectively. DNA with base A inserted in the middle was treated identically. Then, 1 μL of + 5, +11, and + 17 trisite-modified ss-/ds-spDNA (20 nM) was used to replace the previously single-modified spDNA, and the reaction was carried out under identical conditions. 1 μL of + 5, +11, and + 17 trisite-modified ss-/ds-pcDNA (20 nM) was also tested under the same conditions.

### Measurement of crRNA/pcDNA melting temperature

Preparation of crRNA/pcDNA double-stranded hybrid: In a 200 μL PCR tube, pcDNA (4.5 μL, 28 μM), crRNA (5.3 μL, 28 μM), and NEBuffer 4 (5 μL, 10×) were combined, and the total volume was adjusted to 50 μL with deionized water. The mixture was heated to 95°C for 1 min, then cooled to 65°C at 6°C/min for 1 min, followed by cooling to 55°C at 6°C/min for 1 min, and finally cooled to 37°C at 6°C/min to complete annealing. The double-stranded complex was then stored at 4°C. The procedure was performed on the Q1600 Real-Time Fluorescence PCR Instrument.

Measurement of melting temperature: In 200 μL PCR tubes, the prepared crRNA/pcDNA double-stranded hybrids (2.5 μM) were aliquoted into volumes of 5 μL, 2 μL, and 1 μL, respectively. Subsequently, 0.5 μL of EvaGreen (20×) and 2 μL of NEBuffer 4 (10×) were added to each sample, and the total volume was adjusted to 50 μL with deionized water. Melting temperature was immediately measured at a temperature range from 30°C to 90°C using the Rotor-Gene Q real-time PCR instrument.

### Turn-off/on mode verification

For the turn-on model, 0.4 μL of LbCas12a (1 μM), 2 μL of crRNA (200 nM), and 2 μL of NEBuffer 4 (10×) were combined in a 200 μL PCR tube, and the volume was adjusted to 17 μL with deionized water. The mixture was incubated at 37°C for 15 min. Then, 2 μL of HEX-ssDNA reporter (2.5 μM) and 1 μL of pcDNA (20 nM for low pcDNA, 2.5 μM for high pcDNA) were added. Fluorescence intensity was measured at 37°C immediately using the Rotor-Gene Q real-time PCR instrument (QIAGEN, Hilden, Germany) in the HEX channel(gain = 5). 1 μL of activator (17 nt) was added to the tube after 120 s to both groups (low pcDNA and high pcDNA). Then, it was photoactivated with UV light at 365 nm for 3 min. The entire process was recorded by the Rotor-Gene Q real-time PCR instrument.

For the turn-off model, 2 μL of LbCas12a (100 nM), 2 μL of crRNA (500 nM), and 2 μL of NEBuffer 4 (10×) were combined in a 200 μL PCR tube, and the volume was adjusted to 17 μL with deionized water. The mixture was incubated at 37°C for 15 min. Then, 2 μL of HEX-ssDNA reporter (10 μM) and 1 μL of a short activator (12 nt, 500 nM) were added (gain = 3). The reaction first proceeded for 100 s. Then, 1 μL of pcDNA (2.5 μM) was added to the tube.

### Optimization of system parameters

Activator length was optimized by adding 1 μL of short activators of varying lengths (12–17 nt) to the previously constructed Cas12a reaction system (crRNA-50nM, Cas12a-10nM, 1 × NEBuffer 4). 1 μL of the phosphorothioate-modified activator (500 nM) was added to the system, replacing the original activator to enhance the activating ability of the activator. To optimize activator concentration, 1 μL of short activators at different concentrations (200 nM, 400 nM, 1 μM, and 2 μM) was added to the same reaction system using phosphorothioate-modified activator. 1 μL of pcDNA (20 nM, 40 nM, 50 nM, 100 nM, 200 nM, 400 nM, 1 μM, 2.5 μM, 5 μM, 10 μM, 15 μM, and 20 μM) was added to the same reaction system using phosphorothioate-modified activator. Then, the illumination time was optimized by varying the duration of UV illumination (0, 5, 10, 20, 30, 60, 120, 180, and 300 s).

For the effect of the pcDNA photoproduct, we used the fragments of pcDNA after light lysis at gradient concentrations to replace pcDNA to simulate the effect of photoproduct accumulation in different rounds, and treated carboxyfluorescein-modified (FAM-modified) pcDNA with BHQ1-modified complementary strands under different light exposure times to explore the photolysis limit and efficiency of pcDNA (the emission wavelength is 495 nm and the excitation wavelength is 518 nm). In addition, without adding pcDNA, we irradiated the system with different light exposure times to explore the effects of light exposure on Cas12a and nucleic acid activity.

### Establishment of the hierarchical temporal authorization system

In a 200 μL PCR tube, 2 μL of LbCas12a (100 nM), 2 μL of crRNA (500 nM), and 2 μL of NEBuffer 4 (10×) were combined, and the volume was adjusted to 17 μL with deionized water. The mixture was incubated at 37°C for 15 min. Next, 2 μL of HEX-ssDNA reporter (10 μM) and 1 μL of a short phosphorothioate-modified activator (1 μM) were added for five cycles. To stop the reaction, 1 μL of pcDNA (2.5 μM) was added. After 5 min, UV illumination was applied to reactivate the system. For two rounds of activation, the steps for adding pcDNA and UV illumination were repeated.

### Statistical analysis

All experiments in this study were designed with independent replicates. At least three biological replicates (n ≥ 3) were conducted under each experimental condition to ensure the reliability and reproducibility of the results. Statistical analysis and visualization were achieved with the aid of GraphPad Prism 9.0. For the analyses of “the effect of pcDNA fragment accumulation on Cas12a activity,” “the effect of UV light exposure time on Cas12a and nucleic acids,” and “the fluorescence increase rate under different authorization conditions,” as well as other comparative experiments involving multiple experimental groups and control groups, one-way analysis of variance (One-way ANOVA) was employed to examine the differences in experimental outcomes among the groups.

Through statistical analysis of the hierarchical temporal authorization system at different regulatory stages, we first defined the positive control group (CT): this group contained only the activator (with no pcDNA or UV treatment), maintaining Cas12a in a continuously activated state and serving as the baseline for activity quantification. Building on this positive control, we established an “activated-locked” group suppressing Cas12a *trans*-cleavage activity and locking the system in an inactive state. Subsequently, to validate the reversibility of the system, we introduced regulatory treatments involving alternating application of UV light and pcDNA supplementation. Critically, a control group was retained for each regulatory operation to isolate the effect of the target treatment. One-way analysis of variance (One-way ANOVA) was utilized to statistically evaluate the differences in experimental outcomes across the various states.

## Results and discussion

### Design and operation principle

As shown in Fig. 1C, Cas12a activation was inhibited by an intermediate-modified blocker that dissociates upon UV light exposure, allowing the short activator to rebind. Reinhibition was achieved by replenishing the blocker. This system enabled the *trans*-cleavage activity of Cas12a to be switched on and off cyclically by controlling the input conditions (either a blocker or UV light), achieving precise spatiotemporal regulation of Cas12a activity.

Initially, a shorter DNA strand was used as the activator, with the 3′ end of the crRNA as the toehold region. A longer intermediate-modified blocker, using its toehold region, could replace the activator bound to the crRNA, thereby switching Cas12a activity from on to off. When a photosensitive modification group was introduced, exposure to light of a specific wavelength caused the chemical bonds in the modification group to break, resulting in the cleavage of the blocker and its subsequent dissociation from the crRNA. The free activator then re-bound with the crRNA, reactivating the Cas12a. The activity of Cas12a was monitored through changes in the fluorescence signal of a reporter. The reporter is a 15-nucleotide (15-nt) single-stranded DNA (ssDNA) modified with a fluorophore (HEX) and a quencher (BHQ-1) at 5′- and 3′- end, respectively [28]. HEX was initially quenched by BHQ-1 in the reporter. When Cas12a was activated, its non-specific *trans*-cleavage activity hydrolyzed the reporter, producing fluorescent fragments that increased the fluorescence signal. When Cas12a was inhibited by the blocker, its *trans*-cleavage activity was suppressed, and the fluorescence intensity no longer increased. Upon UV irradiation, Cas12a was reactivated, leading to a renewed increase in fluorescence intensity.

### Feasibility validation and blocked site exploration

Since the discovery of Cas12a, research has primarily focused on targeting the conserved stem-loop structure, where crRNA binds to Cas12a, to achieve fine regulation [29]. However, the variable region has been largely unexplored. Although studies have examined the impact of base insertions in the TS on Cas12a activation [30], there has been no comprehensive optimization or characterization of modification types, sites, or group insertions in the TS.

**Figure 2.**
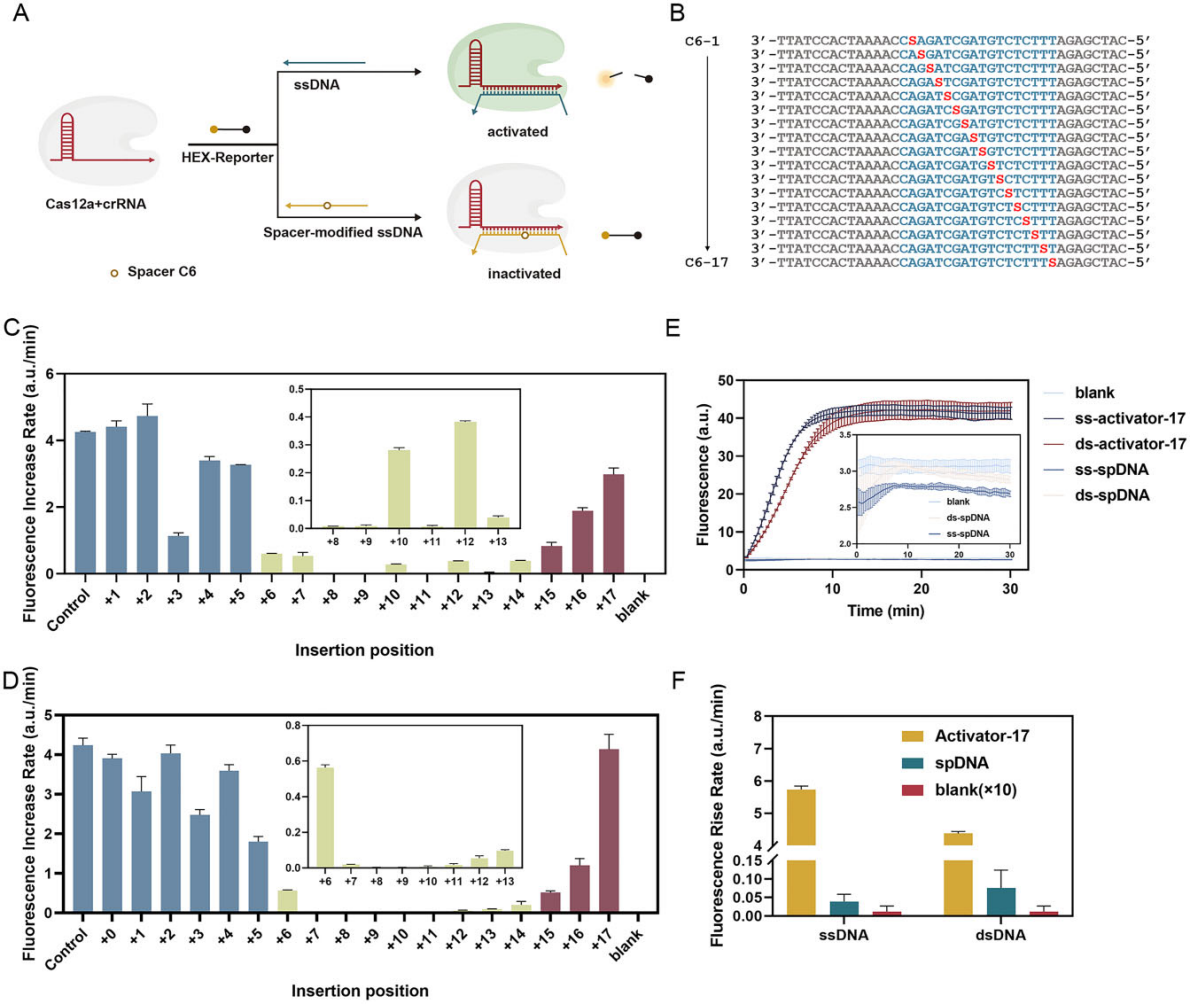
Inhibitory Effects of Different Modifying Groups and Sites on Cas12a Activity. (**A**) Binding of Cas12a-crRNA binary complexes with either exact-match activators (top strand) or intermediate-modified blockers (bottom strand). (**B**) Insertion sites for intermediate modifications in blockers. The S represents Space C6. (**C**) Blocker’s inhibition effect when using Spacer C6 for intermediate modifications. (**D**) Blocker’s inhibition effect when using base A for intermediate modifications. (**E** and **F**) Inhibitory ability of blockers using multibit intermediate modifications. The spDNA represents the blocker with Spacer C6.

We initially used Spacer C6 (Supplementary Fig. S1A), an alkane chain of six methylene groups, as an intermediate modifier to design the blocker. To enhance the blocker’s inhibitory effect on the CRISPR-Cas system for improved regulation, we explored how different modification positions of Spacer C6 affected Cas12a activity, as shown in Fig. 2A and B. The results indicated that Cas12a showed the lowest cleavage activity when modifications were introduced at the middle position (+6 to + 14). In contrast, modifications at the ends of the blocker produced higher cleavage activity (Fig. 2C). This pattern persisted when Spacer C6 was replaced with base A (Fig. 2D).

These results indicate that both bases and modification groups can effectively inhibit Cas12a activity at specific sites. Specifically, when a modification group is inserted between bases at a central position, the resulting bubble creates spatial resistance that interferes with crRNA binding to the target strand, thereby weakening binding affinity.17 While the end position has a small influence on the modification group. To facilitate subsequent photodissociation experiments, we selected DNA with three modified sites with modifications at + 5, +11, and + 17 as the blocker. Next, we evaluated the suppression effect of the multi-site modified blocker. Results showed that both single- and double-stranded blockers with multi-site intermediate modifications effectively inhibited Cas12a (Fig. 2E and F). Thus, the inhibitory effect of intermediate modification depends solely on the modification site and is unaffected by the insertion type or single-/double-stranded status.

### Turn-off and turn-on mode verification

**Figure 3.**
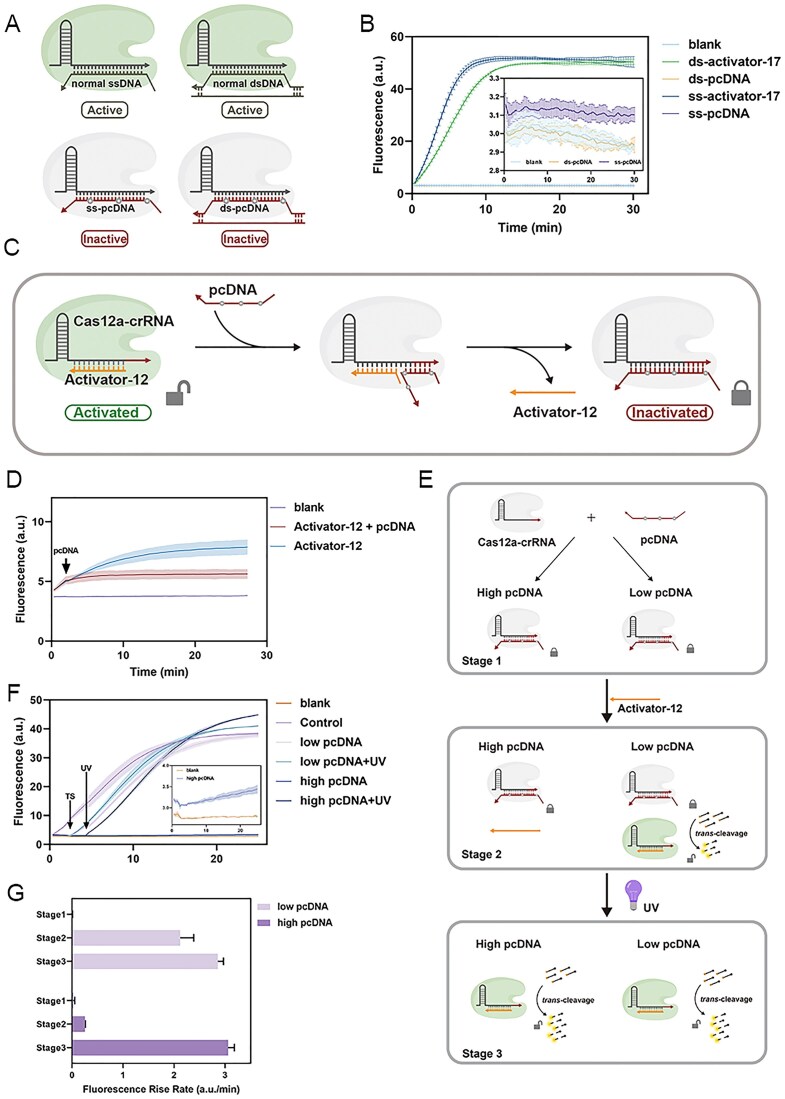
Validation of the Cas12a Activity Switch with pcDNA. (**A**) Activity of Cas12a in different DNA binding to crRNA. (**B**) Activation of Cas12a in the presence of pcDNA (single- and double-stranded forms) with intermediate modifications at positions + 5, +11, and + 17. (**C**-**D**) Turning off Cas12a *trans*-cleavage activity using TMSD reactions. (**E**-**G**) Turning on Cas12a *trans*-cleavage activity using UV irradiation to break the pcDNA. “High pcDNA” and “low pcDNA” represent the system with high concentration and low concentration of pcDNA.

Building on the successful inhibition observed with the modification insert, we replaced the Spacer C6 modification with a 2-nitrobenzyl linker (PC-Linker, Supplementary Fig. S1B), a light-sensitive spacer modification that can be broken under UV light irradiation, generating a photocleavable ssDNA blocker (pcDNA). The relationship between the derived peak (-dF/dT) and temperature presented in Supplementary Fig. S2 indicates that the melting temperature of the double-stranded hybrid formed by pcDNA and crRNA is approximately 50°C. This suggests that these two molecules can stably hybridize under the experimental conditions (37°C). As expected, pcDNA effectively inhibited Cas12a activity in both single- and double-stranded forms (Fig. 3A and B). We further validated the inhibitory effect of the intermediate modification using different crRNA/DNA complementary lengths and sequence variations (BRAF-17 nt, BRAF-20 nt, HCV-17 nt, and HCV-24 nt). The results showed that the inhibition remained effective regardless of length or sequence changes and effectively suppressed Cas12a *trans*-cleavage activity across various concentrations from 1 to 125 nM (Supplementary Fig. S3).

Next, as shown in Fig. 3C, we tested the feasibility of strand displacement between pcDNA and the short activator (12 nt) to switch the CRISPR-Cas system from an active to an inactive state (from on to off). Following the addition of pcDNA, Cas12a’s *trans*-cleavage activity was inhibited, and the reporter strand was no longer cleaved, confirming the successful execution of the strand displacement reaction (Fig. 3D). Additionally, we tested double-stranded DNA as an activator within the system. Although adding pcDNA led to a decrease in fluorescence signal relative to the control, the response was less complete than when a single-stranded activator was used (Supplementary Fig. S4). This is likely because single-stranded pcDNA has more difficulty displacing double-stranded activators than single-stranded ones.

We then investigated whether Cas12a inhibited by pcDNA could be reactivated. As shown in Fig. 3E, we pre-locked the CRISPR-Cas system using two concentrations of pcDNA. In the system pre-locked with a high concentration of pcDNA (125 nM), no significant fluorescence increase was observed after adding the activator, indicating that all Cas12a-crRNA binary complexes were occupied by pcDNA, preventing activator binding and fully inhibiting the system. In contrast, in the system pre-locked with a low concentration of pcDNA (1 nM), only a portion of the binary complexes was occupied, and adding the activator resulted in a clear fluorescence increase, indicating an incompletely off state. Following this, we exposed both systems to 365 nm UV light. In the system with low-concentration pcDNA, only a slight increase in fluorescence rate was observed after irradiation. In the system with high-concentration pcDNA, however, fluorescence increased rapidly to levels comparable to the positive control, indicating that UV exposure cleaved pcDNA, freeing crRNA from inhibition. Then, the activator in the system promptly bound to the crRNA, forming a ternary complex (Cas12a-crRNA-activator) and restoring Cas12a activity (Fig. 3F and G). These results demonstrated the feasibility of reactivating the CRISPR-Cas system from off to on state using UV light in a pcDNA-locked system. For the double-stranded activator system, we also found an increase in the fluorescence signal after the UV light was used, demonstrating that it is feasible to use the double-stranded activator in the “Turn on” model. (Supplementary Fig. S5).

### Optimization of system parameters

**Figure 4.**
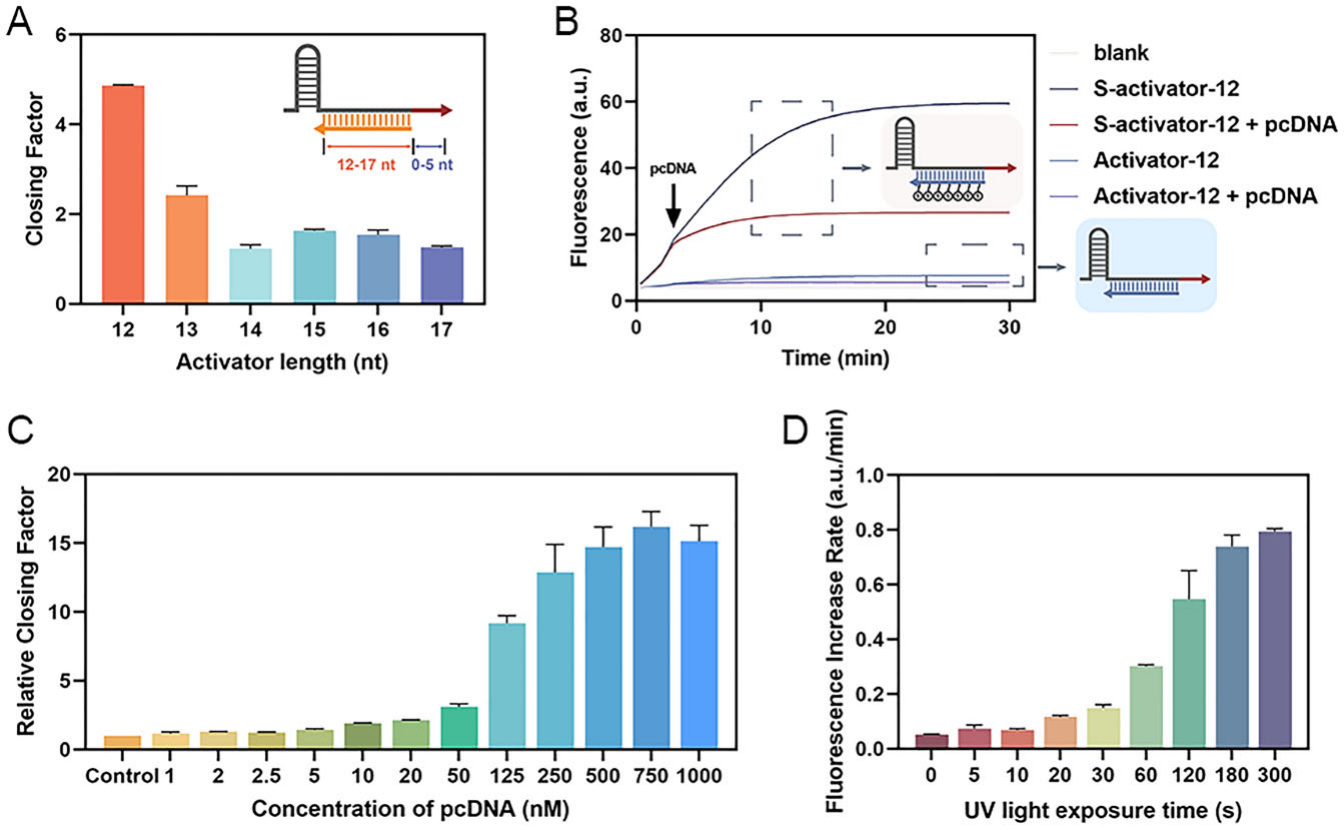
Optimization of conditions for enhanced performance. (**A**) Changes in the closing factor after the addition of pcDNA for systems using activators of different lengths. (**B**) Effect of regulation of Cas12a *trans*-cleavage activity when using phosphorothioate-modified activator. (**C**) The inhibitory effect of different concentrations of pcDNA. (**D**) Reactivation effects at different UV light exposure times.

Based on these findings, we optimized the system parameters to enhance the regulatory effect of the intermediate-modified blocker on Cas12a. As shown in Supplementary Fig. S6, we first optimized the length of the short activator. When the activator was 12 nt, the activation effect was limited; however, the addition of pcDNA produced a stronger inhibitory effect. With increased activator length, the positive control group’s fluorescence signal rose markedly, while pcDNA’s inhibitory effect weakened significantly. This reduction in inhibition likely occurs because lengthening the activator shortens the toe region, making strand displacement more challenging. To balance these factors, we defined a parameter, the closing factor (CF = v_control_/v_closed_), in which v_control_ and v_closed_ represent Cas12a cleavage rate without and with pcDNA off, respectively. A higher CF indicates a stronger inhibitory effect of pcDNA. The results indicate that the closing factor is maximized with a 12 nt activator (Fig. 4A). To further enhance the activation ability of the 12 nt activator, previous studies [28] suggest that phosphorothioate modification could prevent the ssDNA from being cleaved. We therefore tested a fully phosphorothioate-modified 12 nt activator (S-activator-12) and found that it yielded stronger Cas12a cleavage activity compared to the unmodified strand. Additionally, it showed a stronger response to pcDNA, achieving a better inhibitory effect (Fig. 4B). This may be because the loss of single-stranded activator is reduced, more of it is combined with Cas12a, and the activation capacity is enhanced. Meanwhile, the length of the phosphorothioate-modified activator was also optimized (Supplementary Fig. S7).

We then optimized the concentration of the phosphorothioate-modified activator. At 50 nM, the addition of pcDNA produced a more pronounced rate difference (Supplementary Fig. S8). The CF for 50 nM was also good (Supplementary Fig. S9).

Next, we investigated the minimum concentration of pcDNA required for effective inhibition, using a final crRNA concentration of 50 nM (Fig. 4C). When the pcDNA concentration was below 50 nM, inhibition was poor, likely due to insufficient pcDNA to fully bind the crRNA. As a result, complete inhibition was not achieved. At pcDNA concentrations above 50 nM, the inhibition improved significantly, as the excess pcDNA could fully displace the activator, with a marked effect at 125 nM. Although higher concentrations continued to enhance inhibition, 125 nM was selected for subsequent experiments to avoid interference with subsequent light-based reactivation.

Finally, we optimized the time for reactivating the system with UV light exposure. As shown in Fig. 4D and Supplementary Fig. S10, after 180 s, the pcDNA was nearly fully cleaved by UV light and dissociated from the crRNA, allowing the activator to rebind with Cas12a-crRNA, resulting in a fast fluorescence increase. Thus, 180 s was selected as the reactivation time for subsequent experiments.

However, we observed that the fluorescence increase rate slowed down following the reactivation of UV light, which indicates a reduction in the cleavage efficiency of the system. The potential reasons for this phenomenon include the accumulation of pcDNA photoproducts and the limited photolysis efficiency of pcDNA. Additionally, we investigated the effects of UV light on Cas12a and nucleic acids, and no significant impacts were detected (Supplementary Fig. S11). The results indicated that the accumulation of pcDNA photolysis products during a single regulation cycle did not significantly affect Cas12a activity. As the number of simulated regulation cycles increased (with accumulation of pcDNA photolysis fragments), Cas12a’s cleavage rate gradually decreased, which was consistent with the general pattern that cumulative byproducts may interfere with enzymatic reactions over multiple cycles. For the maximum amount of pcDNA cleavage under UV light, we used FAM-modified pcDNA paired with a BHQ1-modified complementary strand, measured fluorescence recovery under different UV light exposure times to investigate pcDNA’s photolysis efficiency. The result showed that pcDNA was not completely cleaved under the experimental conditions. This incomplete cleavage, when accumulated over multiple cycles, might also have contributed to the reduction in Cas12a’s *trans*-cleavage rate.

### Establishment of the hierarchical temporal authorization system

**Figure 5.**
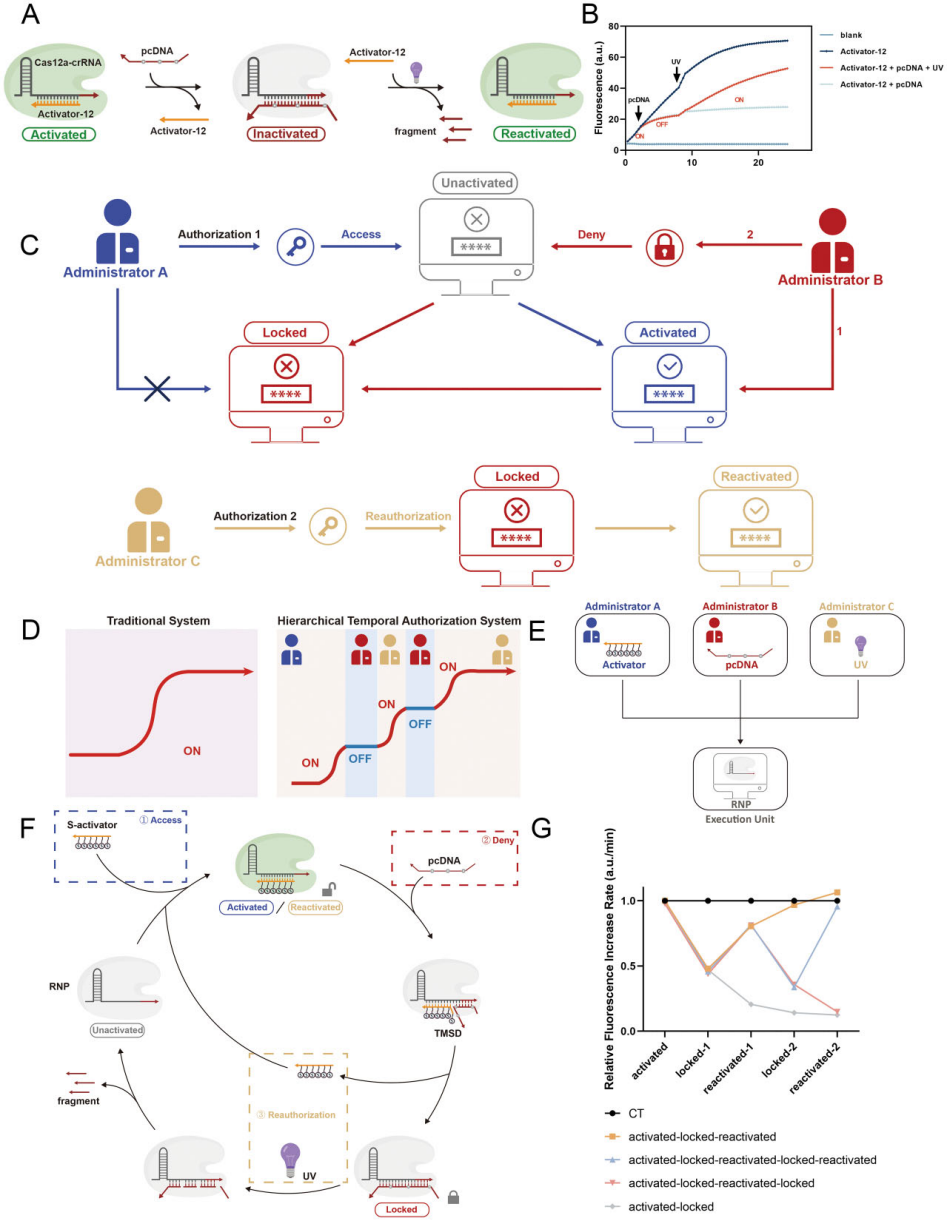
Construction of the hierarchical temporal authorization system. (**A**) A combination of “Turn-off” and “Turn-on” models. (**B**) The inhibition effect of pcDNA and the reactivation effect of UV light throughout the process. (**C**) Schematic of the hierarchical temporal authorization system. (**D**) Comparison of the traditional DNA cryptographic strategies and the hierarchical temporal authorization system. (**E**) Schematic diagram of different levels of administrators. (**F**) Molecular implementation of the hierarchical temporal authorization system. (**G**) Changes in the relative rate of fluorescence increase in authorization.

First, we utilized the optimized results to integrate the “Turn-off” and “Turn-on” processes (Fig. 5A). The results (Fig. 5B and Supplementary Fig. S12) demonstrated that, upon the addition of pcDNA, the *trans*-cleavage activity of Cas12a was inhibited, leading to a deceleration in the fluorescence signal increase due to the strand displacement reaction. This indicated that the “Turn-off” mode proceeded efficiently. Subsequently, UV irradiation was applied, resulting in the cleavage of pcDNA and the release of crRNA, which allowed the activator to rebind to crRNA. Consequently, Cas12a, which had been suppressed by pcDNA, was reactivated, leading to a resumed fluorescence signal increase at a higher rate. This confirmed that the “Turn on” mode also proceeded smoothly. In the absence of UV irradiation, pcDNA continued to bind to crRNA, replacing the short activator and further slowing the fluorescence signal rise rate. In addition, we performed dynamic calculations for the turn-off and turn-on modes. The variation trend of the actual measured rate was consistent with the simulation results (Supplementary Fig. S13, Supplementary Fig. S14, and the section “Kinetics Calculations and Simulation” in Supporting Information). Therefore, we successfully achieved a controllable cascade of “Turn-off” and “Turn-on” modes, enabling the reversible regulation of Cas12a *trans*-cleavage activity. Additionally, we evaluated the combination of the “Turn on” and “Turn off” modes using phosphorothioate-modified double-strand activators and observed that the inclusion of pcDNA did not fully suppress the activity of Cas12a. This suggests that the regulatory effect of double-strand activation following phosphorothioate-modified is less effective compared to single-strand activation (Supplementary Fig. S15).

Most cryptographic strategies rely on a unidirectional response to external stimuli. However, this simple response mechanism offers limited security and does not easily integrate with other biochemical responses, constraining its capacity for more complex functions. [31] To enhance the security of cryptographic strategies, more robust schemes are required. In complex cryptographic applications, administrators have varying levels of permissions, accessible only in a specific sequence. Therefore, we developed a hierarchical temporal authorization system incorporating the Cas12a precision modulation strategy (i.e. the authorization process proceeds in a specific sequence).

As shown in Fig. 5C, Administrator A has a permanent activation authority (blue path). The introduction of Administrator B can terminate the operation of the system accessed by Administrator A (red path 1). Administrator B can also directly lock the system and deny Administrator A’s continued access (red path 2). To reuse the locked system, we designed a higher-level Administrator C, who can cancel the lockdown caused by Administrator B and thus assist Administrator A in restarting the system’s operation.

Traditional single-use cryptographic systems, which lack flexibility in altering the system’s operational state after password entry, are unsuitable for complex reaction networks. As illustrated in Fig. 5D, the hierarchical temporal authorization system allows for compatibility with multiple reaction processes each time the system is turned off, providing enhanced operability and security over traditional one-time, permanent authorization systems. In this framework, shown in Fig. 5E, the Cas12a-crRNA binary complex functions as the execution unit of the security system, while the activator, pcDNA, and UV light serve as three hierarchical administrators (A, B, and C, respectively). The operational principles of this hierarchical temporal authorization system are detailed in Fig. 5F.

First, we verified the “long-term authorization” model (black line in Fig. 5G). Results indicated that when only the activator (Administrator A) was present, the Cas12a *trans*-cleavage activity was continuously active, leading to a sustained high increasing rate in fluorescence signal. In the “locked” mode (gray line in Fig. 5G), the addition of pcDNA (Administrator B) initiated TMSD, causing the activator to dissociate from the crRNA. As pcDNA effectively inhibits Cas12a activity, the system became locked, and the displaced activator could no longer bind to the binary complex, even though it remained in the system, leading to a sustained slowdown in the fluorescence increasing rate. In the “reactivation” mode (yellow line in Fig. 5G), UV light (Administrator C) was applied to the locked system (pcDNA-crRNA bound state), causing pcDNA to break and dissociate. This allowed the activator to re-bind to the exposed binary complex, reactivating Cas12a’s cleavage activity. We then investigated the recyclability of the hierarchical authorization system. During the second round of authorization, the system promptly shut down upon the addition of pcDNA (Administrator B), resulting in a significant slowdown in fluorescence increase rate compared to the control. When UV irradiation was applied, the system reactivated, and the fluorescence increase rate accelerated again (blue line in Fig. 5G). The results showed that, after two rounds of authorization cycling, the activity of Cas12a was restored to 95.4% of the positive control. In contrast, the system was deactivated during the first (gray line in Fig. 5G) and second (pink line in Fig. 5G) rounds and remained at only 12.4% and 14.8% of the activity of the positive control, respectively. This trend was also evident in the visual changes in fluorescence signals (Supplementary Fig. S16). Meanwhile, we conducted a statistical analysis of the fluorescence rise rate under different states (Supplementary Fig. S17). These findings demonstrated that the proposed strategy enables multiple rounds of authorization and repeated cycles within a DNA coding framework.

The hierarchical temporal authorization system we developed offers enhanced security and programmability, representing an advancement over traditional cryptographic strategies.

## Conclusion

In this study, we achieved precise control over the CRISPR-Cas12a system by designing targeted modifications on the crRNA-complementary DNA strand. We utilized TMSD reactions to deactivate the active *trans*-cleavage function of Cas12a through an intermediate-modified blocker. Additionally, the UV light cleaved the blocker to reactivate Cas12a. This UV-TMSD-driven “on-off-on” switch provides precise control over Cas12a activation and deactivation. Meanwhile, we compared this study with the existing strategies, and this study has obvious advantages in response time and operability for the regulation of Cas12a (Supplementary Table S3). One of the most promising applications of this strategy is the construction of a hierarchical temporal authorization system. In this system, various regulatory “layers” or “permissions” can be configured to allow or restrict access to specific Cas12a functions based on particular conditions or stimuli, such as blocker or light exposure. This study thus offers a proof-of-concept for employing CRISPR-Cas systems in temporally controlled, multi-layered biochemical circuits. This approach could be enhanced with DNA logic circuits, enabling biochemical networks that respond to specific stimuli and mimic cell computations, with applications in biosensors, drug delivery, and programmable therapies.

## Supplementary Material

gkaf1178_Supplemental_Files

## Data Availability

The data underlying this article are available in the article and in its online supplementary material.
