# Supplementary material for: Photocontrolled dissociation and toehold-mediated strand displacement-based synergistic regulation of CRISPR-Cas12a
Source: Nucleic Acids Res. 2025 Nov 8;53(20):gkaf1178. doi: 10.1093/nar/gkaf1178 (PMC12596738; doi:10.1093/nar/gkaf1178)
Supplement: gkaf1178_Supplemental_Files [file gkaf1178_supplemental_files.pdf]

Supporting Information

Photocontrolled dissociation and toehold-mediated strand displacement-based synergistic regulation of CRISPR-Cas12a

Jia-ni Wu[1], Changjiang Li[1], Zhujun Liu[1], Xiaolong Li[1], Jiarun Wang[1], Xiaoding Lou[2], Fan Xia[2], Jun Dai[1,*], Tongbo Wu[1,*]

[1] School of Pharmacy, and Department of Obstetrics and Gynecology, National Clinical Research Center for Obstetrics and Gynecology, Tongji Hospital, Tongji Medical College, Huazhong University of Science and Technology, Wuhan, 430030, China

[2] State Key Laboratory of Biogeology and Environmental Geology, Faculty of Materials Science and Chemistry, China University of Geosciences, Wuhan, 430074, China

* To whom correspondence should be addressed. Email: jundai@tjh.tjmu.edu.cn (J. Dai), wutongbo@hust.edu.cn (T. Wu)

Supplementary Tables

Table S1 Oligonucleotide sequences used in this work

Name	DNA sequence (5'-3')
**Reporter for all experiments**	
Reporter-HEX	HEX-TTTTTTTTTTTTTTT-BHQ1
**RNA sequences used in the experiment**	
crRNA-BRAF-17	UAAUUUCUACUAAGUGUAGAUGUCUAGCUACAGAGAAA
crRNA-BRAF-20	UAAUUUCUACUAAGUGUAGAUGUCUAGCUACAGAGAAAUCU
crRNA-HCV-17	UAAUUUCUACUAAGUGUAGAUCUCAGGGCGGACGAGGU
crRNA-HCV-24	UAAUUUCUACUAAGUGUAGAUCUCAGGGCGGACGAGGUUUAGGAU
**DNA sequences used in the experiment**	
Activator-17 (BRAF)	CATCGTTTCTCTGTAGCTAGACCAAAATCACCTATT
Activator-20 (BRAF)	CATCGAGATTTCTCTGTAGCTAGACCAAAATCACCTATT
Complementary chains -17 (BRAF)	AATAGGTGATTTTGGTCTAGCTACAGAGAAATCTCGATG
Activator-17 (HCV)	ACCTCGTCCGCCCTGAG
Activator-24 (HCV)	ATCCTAAACCTCGTCCGCCCTGAG
C6-1	CATCGAGATTTCTCTGTAGCTAGA/iSp6/CCAAAATCACCTATT
C6-2	CATCGAGATTTCTCTGTAGCTAG/iSp6/ACCAAAATCACCTATT
C6-3	CATCGAGATTTCTCTGTAGCTA/iSp6/GACCAAAATCACCTATT
C6-4	CATCGAGATTTCTCTGTAGCT/iSp6/AGACCAAAATCACCTATT
C6-5	CATCGAGATTTCTCTGTAGC/iSp6/TAGACCAAAATCACCTATT
C6-6	CATCGAGATTTCTCTGTAG/iSp6/CTAGACCAAAATCACCTATT
C6-7	CATCGAGATTTCTCTGTA/iSp6/GCTAGACCAAAATCACCTATT
C6-8	CATCGAGATTTCTCTGT/iSp6/AGCTAGACCAAAATCACCTATT
C6-9	CATCGAGATTTCTCTG/iSp6/TAGCTAGACCAAAATCACCTATT
C6-10	CATCGAGATTTCTCT/iSp6/GTAGCTAGACCAAAATCACCTATT
C6-11	CATCGAGATTTCTC/iSp6/TGTAGCTAGACCAAAATCACCTATT
C6-12	CATCGAGATTTCT/iSp6/CTGTAGCTAGACCAAAATCACCTATT
C6-13	CATCGAGATTTC/iSp6/TCTGTAGCTAGACCAAAATCACCTATT
C6-14	CATCGAGATTT/iSp6/CTCTGTAGCTAGACCAAAATCACCTATT
C6-15	CATCGAGATT/iSp6/TCTCTGTAGCTAGACCAAAATCACCTATT
C6-16	CATCGAGAT/iSp6/TTCTCTGTAGCTAGACCAAAATCACCTATT
C6-17	CATCGAGA/iSp6/TTTCTCTGTAGCTAGACCAAAATCACCTATT
spDNA	CATCGAGA/iSp6/TTTCTC/iSp6/TGTAGC/iSp6/TAGACCAAAATCACCTATT
17-0-A	CATCGAGATTTCTCTGTAGCTAGACACAAAATCACCTATT
17-1-A	CATCGAGATTTCTCTGTAGCTAGAACCAAAATCACCTATT
17-2-A	CATCGAGATTTCTCTGTAGCTAGAACCAAAATCACCTATT
17-3-A	CATCGAGATTTCTCTGTAGCTAAGACCAAAATCACCTATT
17-4-A	CATCGAGATTTCTCTGTAGCTAAGACCAAAATCACCTATT
17-5-A	CATCGAGATTTCTCTGTAGCATAGACCAAAATCACCTATT
17-6-A	CATCGAGATTTCTCTGTAGACTAGACCAAAATCACCTATT

17-7-A	CATCGAGATTTCTCTGTAAGCTAGACCAAAATCACCTATT
17-8-A	CATCGAGATTTCTCTGTAAGCTAGACCAAAATCACCTATT
17-9-A	CATCGAGATTTCTCTGATAGCTAGACCAAAATCACCTATT
17-10-A	CATCGAGATTTCTCTAGTAGCTAGACCAAAATCACCTATT
17-11-A	CATCGAGATTTCTCATGTAGCTAGACCAAAATCACCTATT
17-12-A	CATCGAGATTTCTACTGTAGCTAGACCAAAATCACCTATT
17-13-A	CATCGAGATTTCATCTGTAGCTAGACCAAAATCACCTATT
17-14-A	CATCGAGATTTACTCTGTAGCTAGACCAAAATCACCTATT
17-15-A	CATCGAGATTATCTCTGTAGCTAGACCAAAATCACCTATT
17-16-A	CATCGAGATATTCTCTGTAGCTAGACCAAAATCACCTATT
17-17-A	CATCGAGAATTTCTCTGTAGCTAGACCAAAATCACCTATT
pcDNA (BRAF)	CATCGAGA/iPCLink/TTTCTC/iPCLink/TGTAGC/iPCLink/TAGACCAAAATCACCTATT
pcDNA (HCV)	CATCGAGAATCCTAA/iPCLink/ACCTCG/iPCLink/TCCGCC/iPCLink/CTGAGCAAAATCACCTATT
Activator-12	CTGTAGCTAGAC
Activator-13	TCTGTAGCTAGAC
Activator-14	CTCTGTAGCTAGAC
Activator-15	TCTCTGTAGCTAGAC
Activator-16	TTCTCTGTAGCTAGAC
Activator-17	TTTCTCTGTAGCTAGAC
S-Activator-12	C*T*G*T*A*G*C*T*A*G*A*C
ds-Activator-12	CTGTAGCTAGACCAAAATCA
ds-Complementary chains-12	TGATTTTGGTCTAGCTACAG
S-ds-Activator-12	C*T*G*T*A*G*C*T*A*G*A*C*C*A*A*A*A*T*C*A

* represents phosphorothioate modification; /iSp6/ and /iPCLink/ represent Spacer C6 and PC Linker modification.

Table S2 Buffer used in experiments

Name	DNA sequence (5'-3')
**Cas12a reaction buffer**	
NEBuffer 4 (1×)	50 mM Potassium Acetate<br>20 mM Tris-acetate<br>10 mM Magnesium Acetate<br>1 mM DTT<br>pH 7.9@25°C
**Cas12a Storage conditions**	
Cas12a Diluent	500 mM NaCl<br>20 mM sodium acetate<br>0.1 mM EDTA<br>0.1 mM TCEP<br>50% Glycerol<br>pH 6 @ 25°C

Table S3 Comparison of different regulation methods for CRISPR-Cas12a system

Strategy	Regulatory time	Activity recovery	Regulatory direction	Circular ability	Limitation	Reference
Light-activated CRISPR-Cas12a for amplified imaging of microRNA	On: 5 min	Unidentified	Turn-on	No	Once the activity of Cas12a is activated, it cannot be regulated.	[1]
Chemical control for crosslinked crRNA	On: ≈10 min<br>Off: 10 min	83.2%	Turn-on/off	No	In the Turn-off mode, crRNA is directly inactivated by chemical reagents and is essentially irreversible	[2]
RNA G-Quadruplex at the 5' end of CRISPR RNA	On: 30 s/10 min<br>Off: 10 min	＞90%	Turn-on/off	Yes	KCl and PDS do not have the ability to be suppressed the system. The external G-tetrahedron is reversible when in turn-on mode, but it depends on the system conditions (not explored).	[3]
Engineering Anti-CRISPR Proteins	On: ＞2 h	66.1%	Turn-on	No	The operation is cumbersome and irreversible	[4]
Photocontrolled dissociation and toehold-mediated strand displacement-based synergistic regulation of CRISPR-Cas12a	On: 3 min<br>Off: 10 min	＞95%	Turn-on and turn-off	Yes	In practical use, sequence design needs to be adjusted according to the target, and more exploration is required for actual application	This study

Supplementary Figures

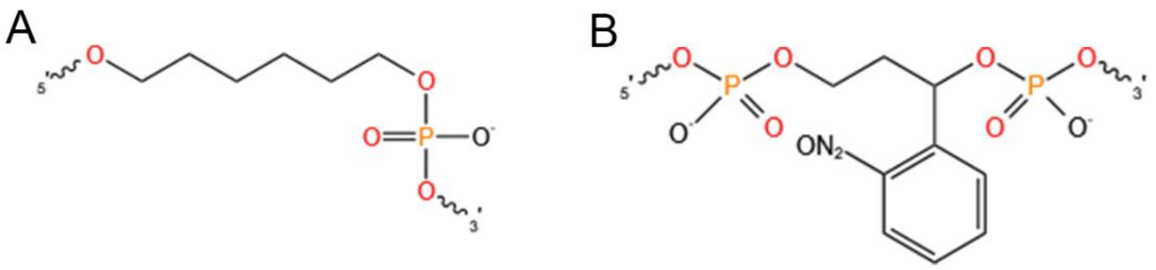

Figure S1 Structure of Spacer C6 (A) and PC Linker (B).

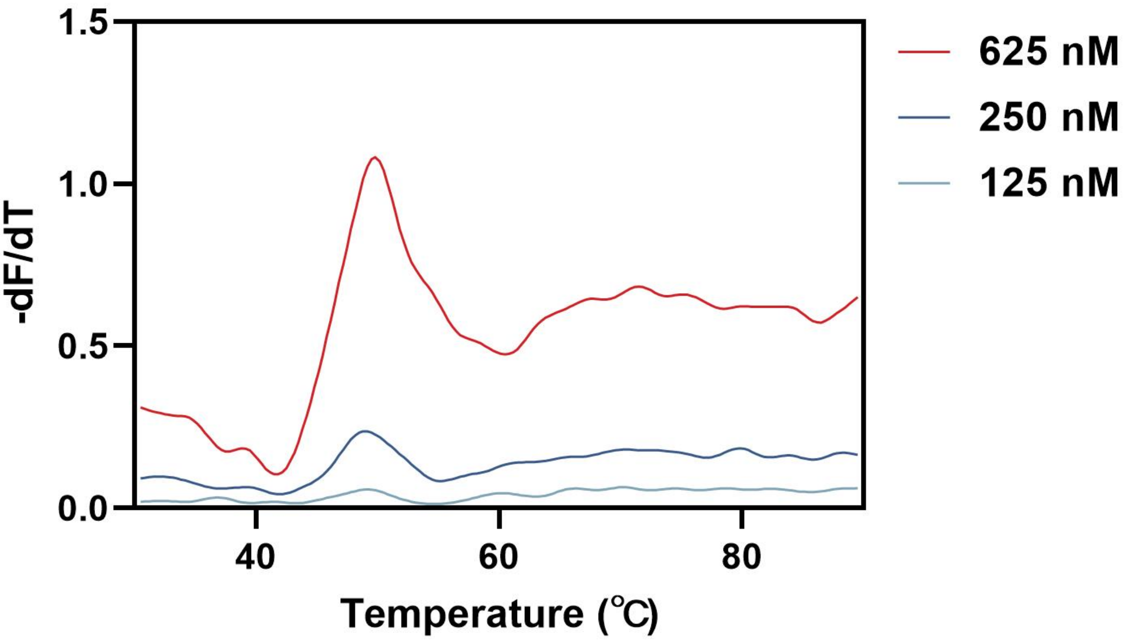

Figure S2 Melting temperature of crRNA and pcDNA. The raw fluorescence data was converted to either the negative first derivative of the fluorescence with respect to temperature (-dF/dT).

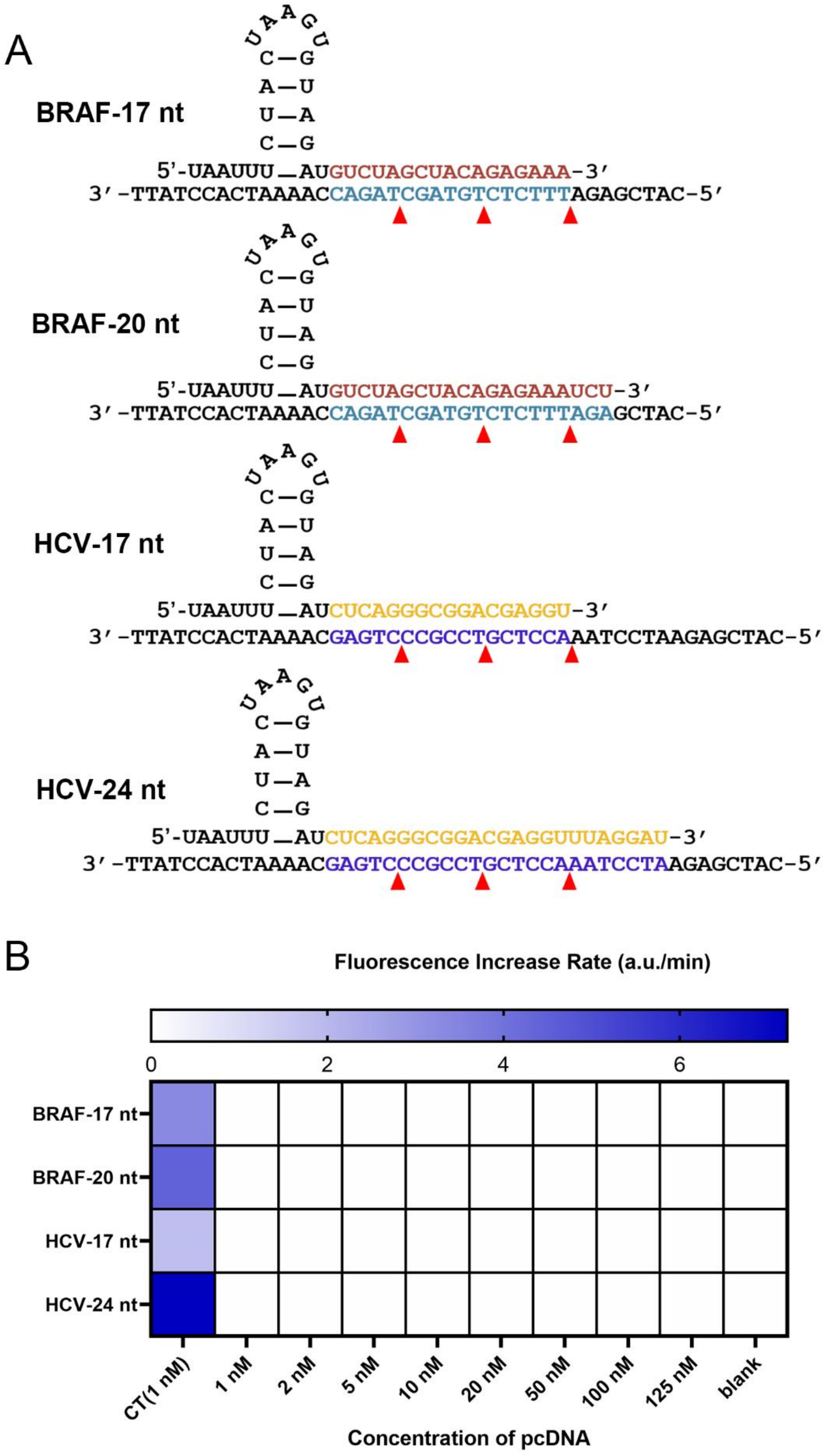

Figure S3 (A) Different sequences and modification sites of pcDNAs. (B) The activation capacity of Cas12a using different pcDNAs as described above.

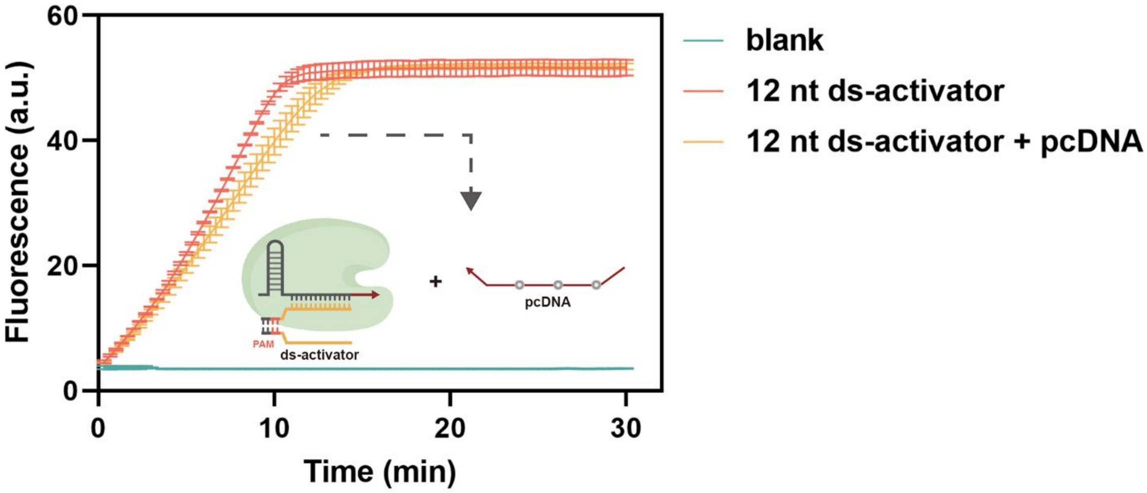

Figure S4 Turning off Cas12a *trans*-cleavage activity using TMSD reactions with double-strand activator (ds-activator).

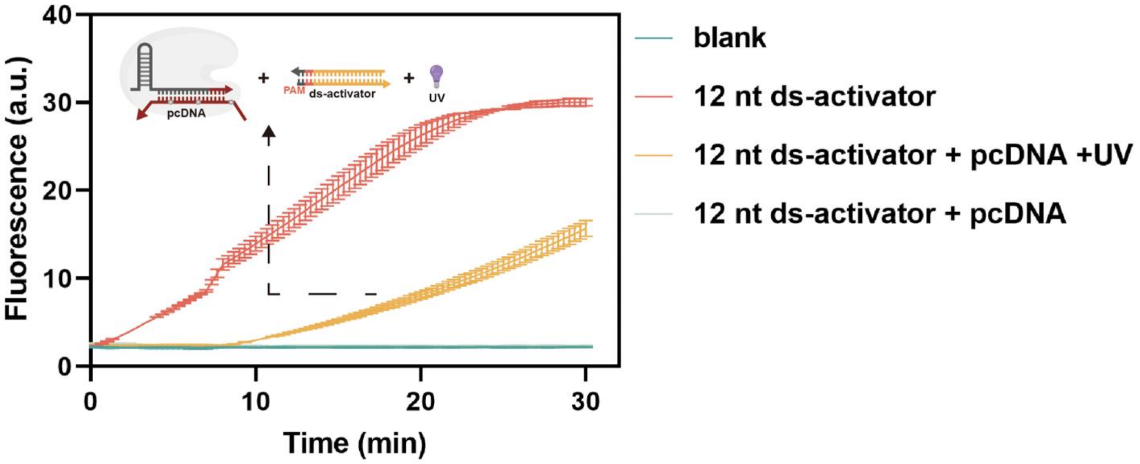

Figure S5 Turning on Cas12a *trans*-cleavage activity using UV irradiation to break the pcDNA with ds-activator.

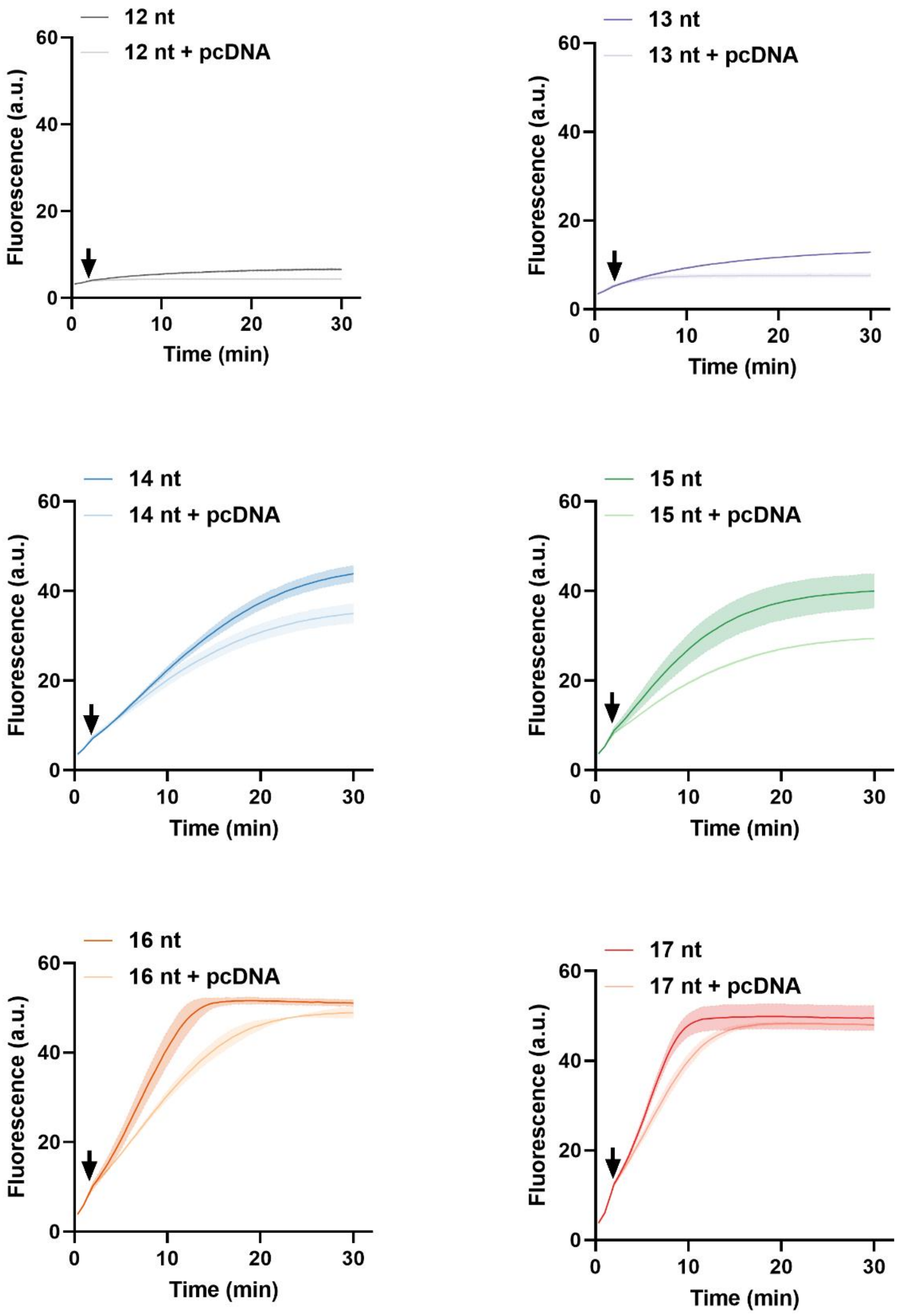

Figure S6 Changes in the fluorescence after the addition of pcDNA for systems using activators of different lengths. The length of 12 to 17 nt corresponds to the toe region of 5 to 0 nt.

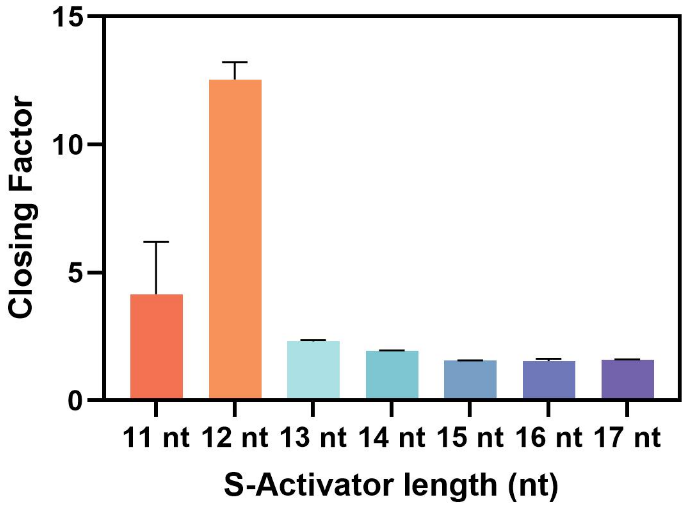

Figure S7 Changes in the closing factor after the addition of pcDNA for systems using phosphorothioate-modified activators of different lengths.

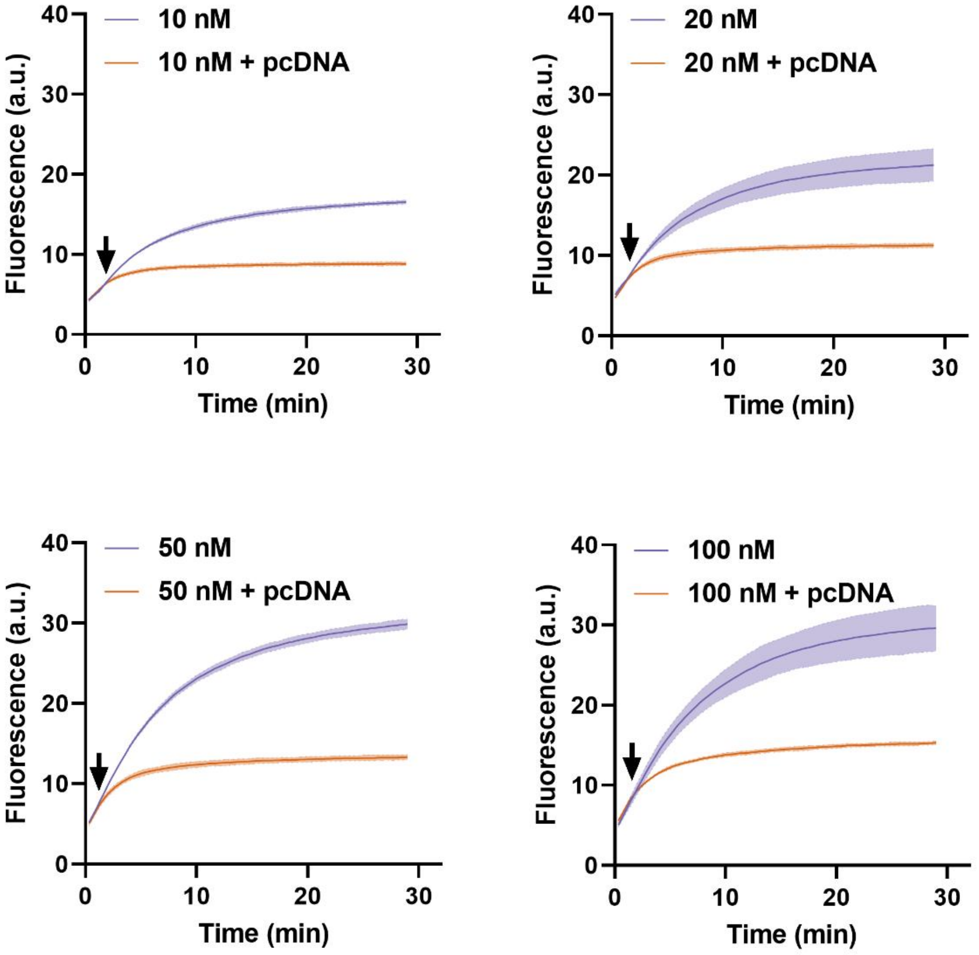

Figure S8 Changes in the rate of fluorescence increase after the addition of pcDNA for systems using activators of different concentrations.

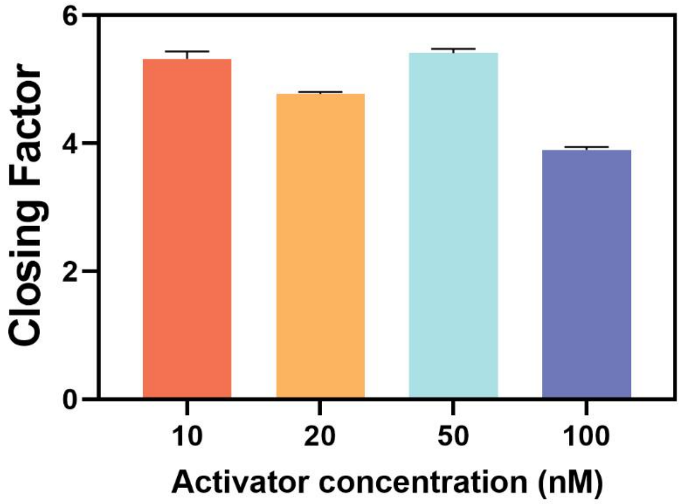

Figure S9 The closing factors of pcDNA when activators of different concentrations are used.

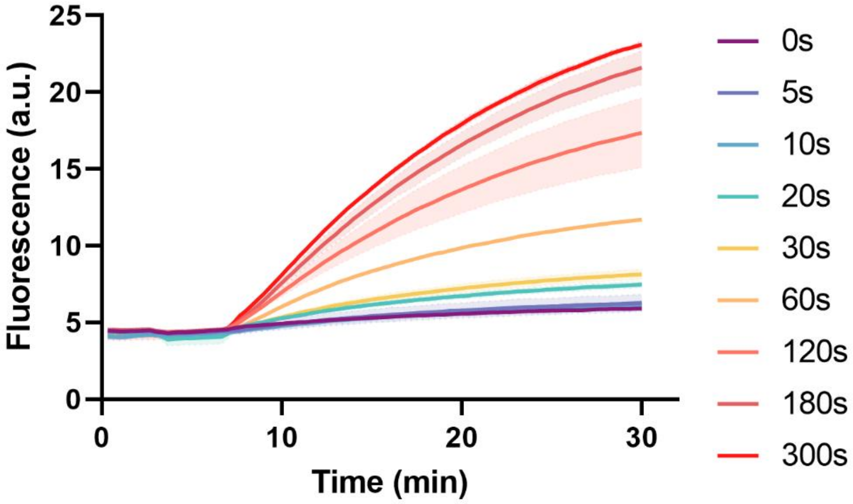

Figure S10 Reactivation effects at different UV light exposure times.

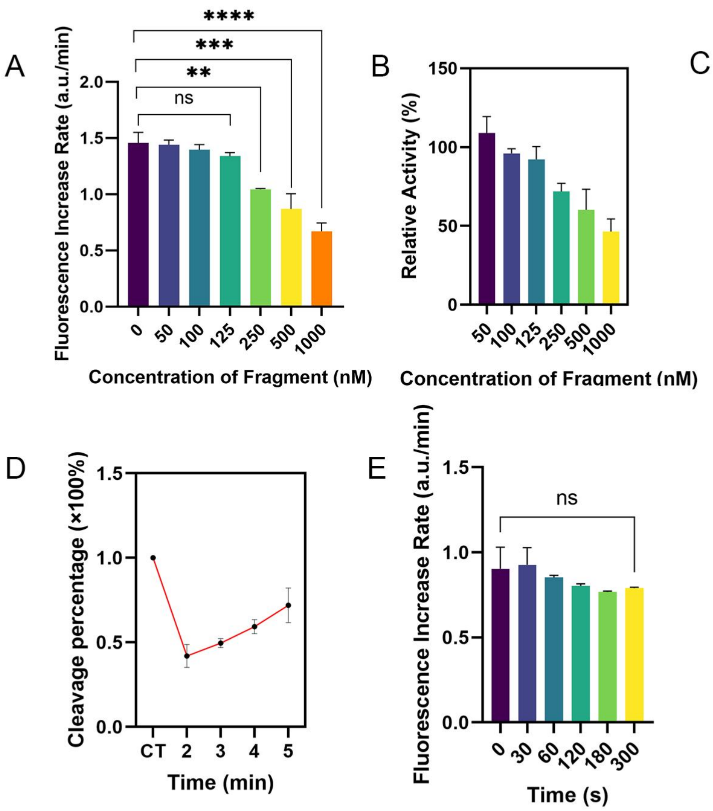

Figure S11 The influence of UV light exposure times on system. (A) The simulation of the accumulation of pcDNA photolysis fragments. (B) The activity of cas12a corresponding to different conditions in (A). (C) The photolysis limit of pcDNA, (D) Cleavage percentage of PC-Linker under different time. (E) The influence of UV light exposure times on the activities of Cas12a and nucleic acids in the system. Error bar, SD, n=3. The data were analyzed using one-way ANOVA. ns means no significance, ** means $p<0.01$, *** means $p<0.001$, **** means $p<0.0001$.

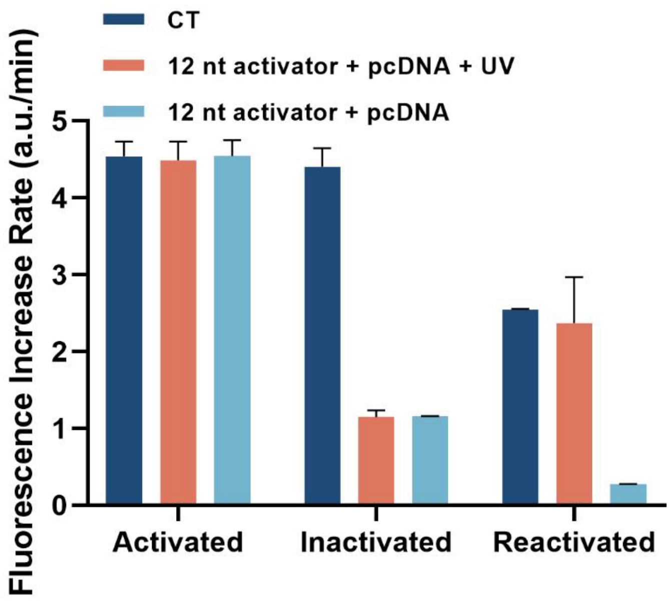

Figure S12 The combination of turn off and turn on modes completely regulating cas12a activity (on to off to on).

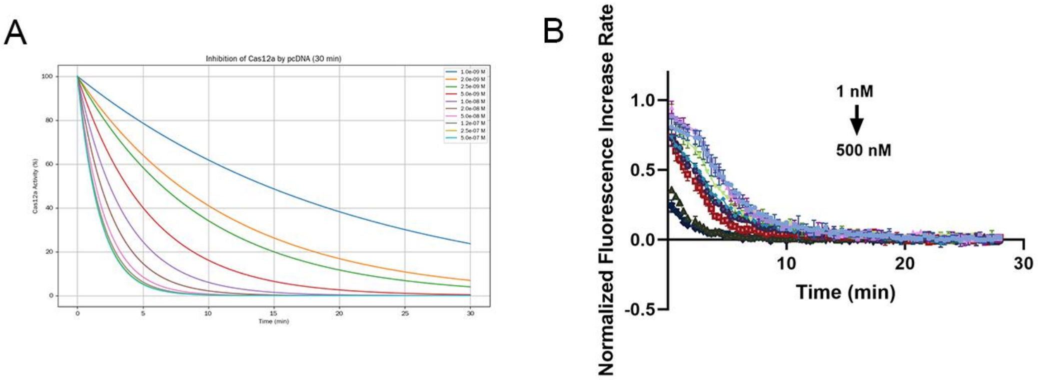

Figure S13 (A) Simulation result of inhibition of Cas12a by pcDNA in turn-off model. (B) The variation of the fluorescence increase rate over time after the addition of pcDNA.

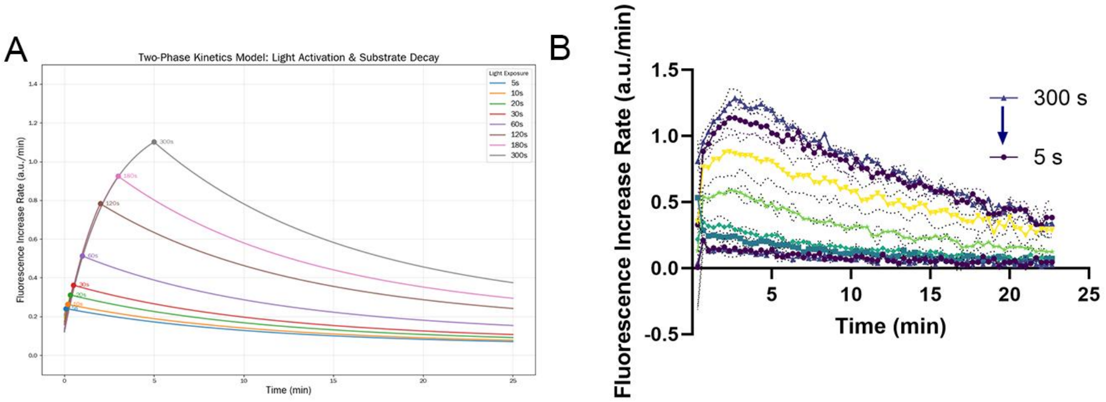

Figure S14 (A) Simulation result of reactivation of Cas12a by UV light in turn-on model. (B) The variation of the fluorescence increase rate over time after the addition of UV light.

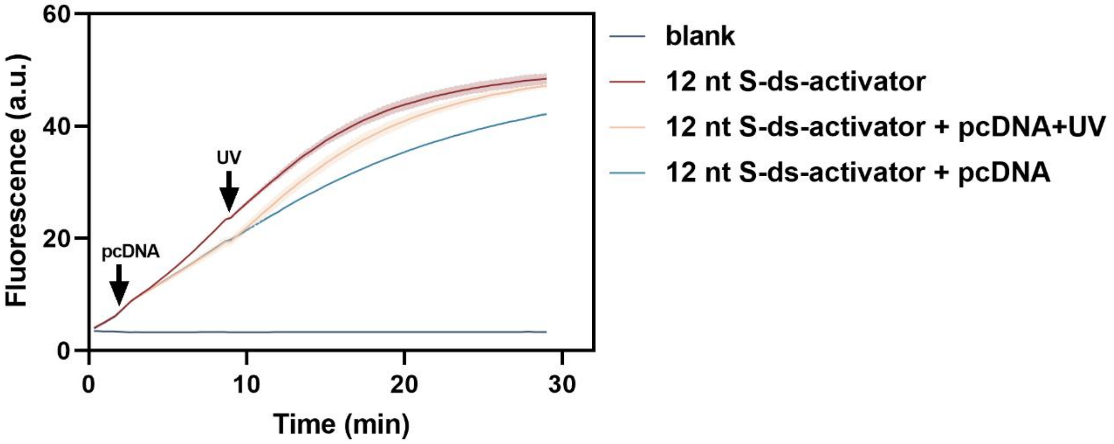

Figure S15 Changes in Cas12a *trans*-cleavage activity in response to various stimuli (pcDNA: inhibition, UV light: reactivation) relative to the positive control.

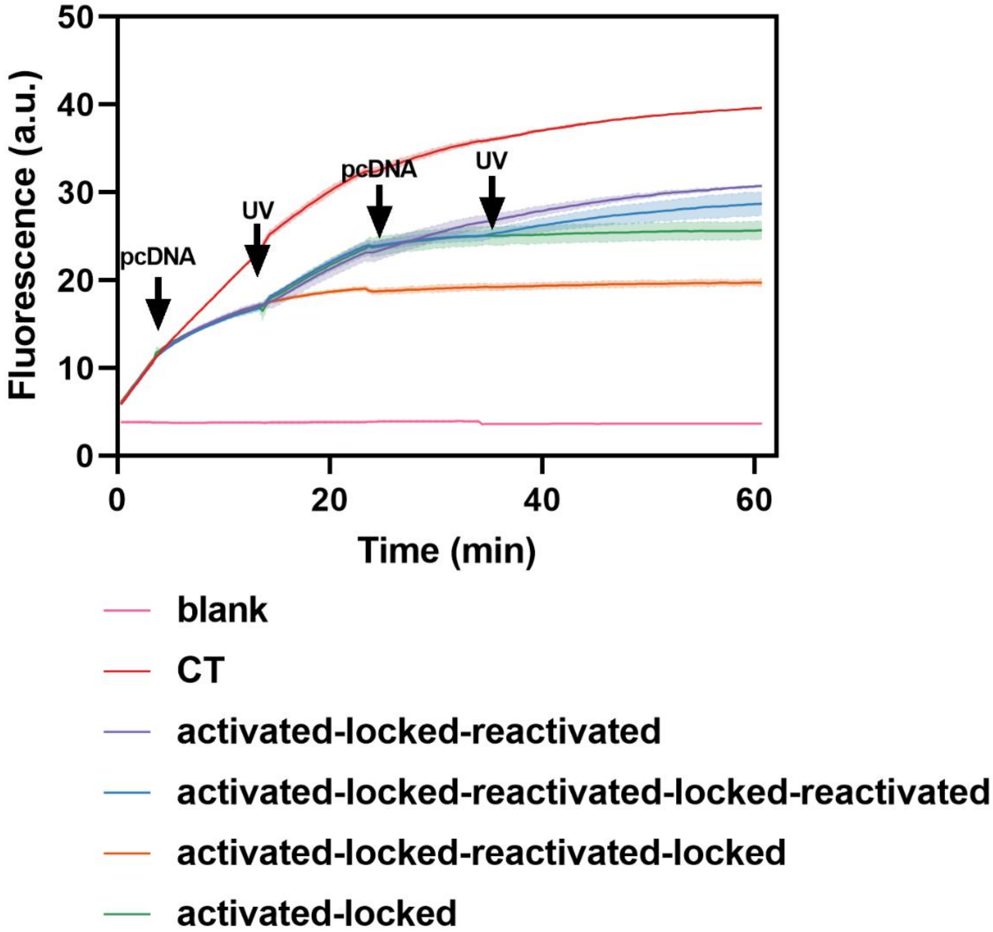

Figure S16 Fluorescence signal changes across multiple regulation cycles.

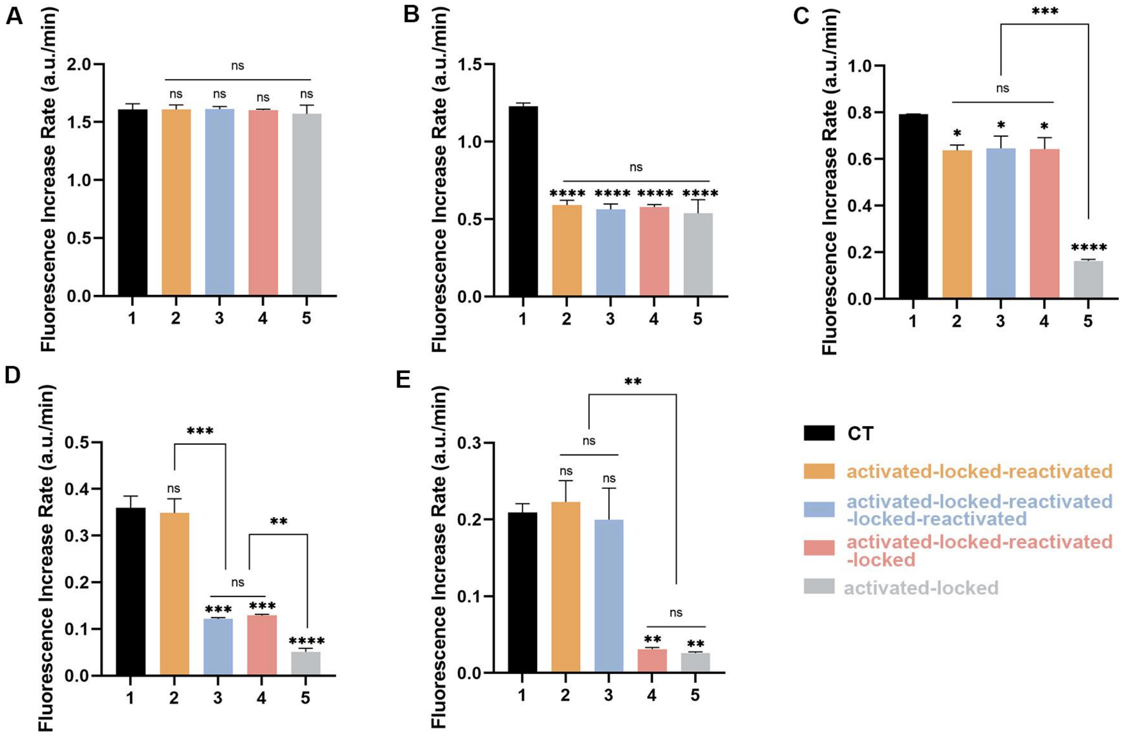

Figure S17 The fluorescence increase rate under different authorization conditions. (A) The first round of activation. (B) The first round of lock. (C) The first round of reactivation. (D) The second round of lock. (E) The second round of reactivation. Error bar, SD, n=3. The data were analyzed using one-way ANOVA. ns means no significance, * means p<0.1, ** means p<0.01, *** means p<0.001, **** means p<0.0001. The marks above the columns indicate the comparison with CT.

Kinetics Calculations and Simulation

To verify the feasibility of the authorization system, we performed dynamic measurements, calculations, and simulations for both the turn-off and turn-on modes.

For the turn-off mode, we first quantified the binding kinetics between Cas12a RNP and the activator, as well as the kinetic process of the strand displacement reaction between pcDNA and the RNP-activator complex. Following the method established by Li et al (5). for calculating Cas12a and RNP kinetics, we monitored the *trans*-cleavage rate as an indicator of Cas12a activity.

crRNA-activator + pcDNA ↔ crRNA- pcDNA + activator, with rate constant k_{on} and k_{off}.

The fluorescence increase rates obtained after treatment with different pcDNA concentrations were normalized:

$$Relative\ rate\ (t) = \frac{v_t - v_{end}}{v_0 - v_{end}},$$

v_0 represented the initial rate, v_t represented the rate at a specific moment, and v_{end} represented the rate when completely suppressed ($v_{end} \approx 0$).

The above formula could be simplified as:

$$Relative\ rate\ (t) = \frac{v_t}{v_0}.$$

(i)At low pcDNA concentrations, "the collision-mediated binding of pcDNA to the Cas12a-crRNA complex" became the rate-limiting step, and the relative rate decayed exponentially over time:

$$Relative\ rate\ (t) = A \cdot e^{-k_{obs} \cdot t} + B$$

k_{obs} was apparent rate constant. A was initial relative rate specific gravity ($A \approx 1$), and B was residual rate specific gravity ($B \approx 0$). Fitted the model to obtain the k_{obs} corresponding to each concentration.

(ii)At high pcDNA concentrations (with rate decay initially fast and then slow), the strand displacement process was completed in two parts:

Fast step: Initial binding of pcDNA to the complex (fast, k_1)

Slow step: Completion of strand displacement (conformation rearrangement, slow, k_2).

The relative rate decayed as a double exponential with time:

$$Relative\ rate\ (t) = A_1 \cdot e^{-k_1 \cdot t} + A_2 \cdot e^{-k_2 \cdot t} + B,\ k_{obs} = x \cdot k_1 + y \cdot k_2$$

During the strand displacement reaction process, k_{obs} first increased with the concentration of pcDNA and then tended to stabilize, which conformed to:

$$k_{obs} = \frac{k_{max} \cdot [pcDNA]}{K_d + [pcDNA]} + k_{background}$$

We fitted the model to obtain the k_{max} and K_d. k_{max} and K_d were 0.6053 min^{-1} and 1.160×10^{-8} M. We further fitted the dynamic curve in the turn-off mode (Figure S13).

For the turn-on mode, we represented the *trans*-cleavage activity of Cas12a by calculating the fluorescence increase rate after different exposure times and conducted a fitting to determine the key kinetic parameters:

The variation of activation rate over time should satisfy:

$$v(t) = v_{max} \cdot \left(1 - e^{-kt}\right),$$

v_{max} represented the maximum rate, and k is the UV light activation rate constant. Through fitting, v_{max} was 1.238 a.u./s and k was 0.006996 s^{-1}.

As the reaction proceeds, the report was gradually consumed and the signal generation rate decreased. We conducted dynamic calculations on the rate decay process and concluded that the decay rate constant k_{decay} was 0.06593. Then, we further fitted the dynamic curve in the turn-off mode (Figure S14).

Reference

[1] Liu H et al., Light-activated CRISPR-Cas12a for amplified imaging of microRNA in cell cycle phases at single-cell levels.Sci. Adv.10,eadp6166(2024).

[2] Lin C, Chen W, Liu L et al. Chemical Control of CRISPR/Cpf1 Editing via Orthogonal Activation and Deactivation of Crosslinked crRNA. Chem. Commun. 2024, 60 (39), 5197–5200.

[3] Huang W, Wang J, Wang C et al. Expanding Cas12a Activity Control with an RNA G-Quadruplex at the 5' end of CRISPR RNA. Adv Sci (Weinh). 2025, 12(7), e2411305.

[4] Kang W, Xiao F, Zhu X et al. Engineering Anti‐CRISPR Proteins to Create CRISPR‐Cas Protein Switches for Activatable Genome Editing and Viral Protease Detection. Angew Chem Int Ed 2024, 63 (16), e202400599.

[5] Li X, Zhu Z, Wu J et al. PAM-free hairpin target binding activates trans-cleavage activity of Cas12a, Nucleic Acids Research, 2025, 53(12), gkaf596.
